# Supplementary material for: Patterns of Intron Gain and Loss in Fungi
Source: PLoS Biol. 2004 Nov 30;2(12):e422. doi: 10.1371/journal.pbio.0020422 (PMC532390; doi:10.1371/journal.pbio.0020422)
Supplement: Table S1 — Also available at http://genes.mit.edu/NielsenEtAl/. (4.3 MB ZIP). [file pbio.0020422.st001.zip › NielsenEtAl/html/71.html]

AN1616.1.NCU07723.1.MG01713.1.FG06217.1


```
 CLUSTAL W (1.82) Multiple Sequence Alignments - Introns Inserted


Sequence 1: NCU07723.1	363 aa
Sequence 2: MG01713.1	379 aa
Sequence 3: FG06217.1	354 aa
Sequence 4: AN1616.1	404 aa
Alignment Length: 410 aa
Number Identitical Residues: 147 aa
Alignment Score (without introns) 8427


MG01713.1 	-MALPMAPPAKSPLGRYRLLSPTAGVRVSPLCLGAMNFGNGW2KAHMGACDQQQTEEILD
NCU07723.1	-MALPMAPPPKGPLGRYRLLSPTASVRVSPLCLGSMNFGDSW2QDYMGECNQETTESILD
FG06217.1 	-MALPAAPPPKSPLGRYRLLSPTASIWVSPICLGTMNFGNAW2KELMSECNQSTAESIMD
AN1616.1  	MSMFPPAPKPPTLLGYHRVLSPSAGVKVSPLCLGAMNFGDAW2KEYMGECNKEQTFALLD
          	   :* ** .   ** :*:***:*.: ***:***:****:.* :  *. *::. :  ::*

MG01713.1 	YFYSQ0GG1NFIDT~ANNYQFEESETWIGEWMKKRGVRDQMV1IATKYTTNYRSGPAGEG
NCU07723.1	FYYEN~GG1NFIDT~ANNYQKEESEKWIGEWMKKRGIRDQL-~-----------------
FG06217.1 	FYYEQ0GG~NFIDS~ANNYQFQETEKWVGEWMSKRGNRDEI-~-----------------
AN1616.1  	AFYEA~GG~NFIDT2ANNYQQEESEKWIGEWLKKRGNRDQMV2IATKYTTGFRTSHRATE
          	 :*.  ** ****: ***** :*:*.*:***:.*** **::   ::. ::.  :.  .  

MG01713.1 	S~IMANYTGNSTKSLRSSIDASLKKLQTEYIDL0LYVHWWDYSTSIPELMQSLNQLVAAG
NCU07723.1	E1IMANFGGNGTKSMRVSVEASLKKLQTDYIDL0LYVHWWDFATSIPELMQSLNQLVTSG
FG06217.1 	V1LATKYTTN----FRAGPGSPN--------IM0LYVHWYDGNTTIPELMQSLNQLVLSG
AN1616.1  	P~LQSNFVGNSFKSMRVSVDNSLRKLQTDYIDI~LYLHWWDFTTSVEEVMHGLNSLVTAG
          	  : :::  *. .::* .   .  . .:.   : **:**:*  *:: *:*:.**.** :*

MG01713.1 	KVLYLGISDAPAWVVS~KANEYARNHGLRQFSVYQGKWSAASRDFERDIIPMAKDEG0MA
NCU07723.1	KVLYLGVSDTPAWVVS~KANQYARDHALRQFCIYQGRWSAASRDFERDIIPMCRDEG~MG
FG06217.1 	KVLHLGISDTPAWIVS2KANEYARCNGLRQFSVYQGRWSAASRDFEREIIPMCRAEG~MG
AN1616.1  	KVLYLGVSDTPAWVVV~KANDYARAHGLKPFSVYQGKWNAAYRDMEREIVPMCRDQG~MG
          	***:**:**:***:*  ***:*** :.*: *.:***:*.** **:**:*:**.: :* *.

MG01713.1 	LAPWGALGSGNFKTEEQRKN-TDGRRSR--PATEADIKISQVLETIAKRKGSIITSVA--
NCU07723.1	LAPWGALGGGSFKSEEQRQQQANEGRKI--KATEEQIKISRALEKIAQRKNTAITSVA--
FG06217.1 	IAPWGSLGGGKFKSEEQRNA--QDGRKV--VASEQEIRTSRVLESISNRKTTLITSVA--
AN1616.1  	IAPWAPLGGGKFKSAEARKAASSGGSNRGAEMSESDIRISDALEKIAERKKTTLHAIVSH
          	:***..**.*.**: * *:  :.   . .:  :* :*: * .**.*::** : : ::.: 

MG01713.1 	------------------LAYVMHKSPYVFPIV0GGRTVDHLKQNIEALALELNSEEIAE
NCU07723.1	------------------LAYVMHKAPYVFPIV~GGRSVEHLKGNIEALTLALSDKDIDE
FG06217.1 	------------------LAYVMHKTSYVFPII~GGRKIEHLKANIQALTLKLTQEDIQE
AN1616.1  	PCQYPYLYSITDQCPCQALAYVMHKTPNVFPIV~GQRKIEHLKANIEALSISLSDADMDE
          	... .   : :......:*******:. ****: * *.::*** **:**:: *.. :: *

MG01713.1 	IEGAVPFDLGFPHVMLWGEKA-PENRQQVWLVNMGGQFDYVPEPK0P----IPASKGGV-
NCU07723.1	IESAVPFDIGFPSSFLYGEKV-PAHPSQVWLMGMAGTYDYVPVPK~PI---VPAAENGQ-
FG06217.1 	IDSAVKFDPGFPHDFLYRKGQ-TIGGPDVWLLQMGGTVDHIQPSK0YFGREMHPSRDEEL
AN1616.1  	IDGATEFDVGFPMSFIFREFTGRNTAADVWLTKASALIDAPPQPG~P----VRHREAER-
          	*:.*. ** ***  ::: :  .     :***   ..  *    .       :   .    

MG01713.1 	-
NCU07723.1	-
FG06217.1 	F
AN1616.1  	-
          	
```
